# Age at onset of major depressive disorder in Han Chinese women: Relationship with clinical features and family history^☆^

**DOI:** 10.1016/j.jad.2011.06.056

**Published:** 2011-12

**Authors:** Fuzhong Yang, Yihan Li, Dong Xie, Chunhong Shao, Jianer Ren, Wenyuan Wu, Ning Zhang, Zhen Zhang, Ying Zou, Jiulong Zhang, Dongdong Qiao, Chengge Gao, Youhui Li, Jian Hu, Hong Deng, Gang Wang, Bo Du, Xumei Wang, Tiebang Liu, Zhaoyu Gan, Juyi Peng, Bo Wei, Jiyang Pan, Honghui Chen, Shufan Sun, Hong Jia, Ying Liu, Qiaoling Chen, Xueyi Wang, Juling Cao, Luxian Lv, Yunchun Chen, Baowei Ha, Yuping Ning, YiPing Chen, Kenneth S. Kendler, Jonathan Flint, Shenxun Shi

**Affiliations:** a Shanghai Mental Health Centre, Shanghai Jiao Tong University, No. 600 South Wan Ping Road, Shanghai 200030, PR China; b Wellcome Trust Centre for Human Genetics, Oxford OX3 7BN, UK; c Department of Psychiatry, Huashan Hospital, Fudan University, No. 12 Middle Wulumuqi Road, Shanghai, 200040, PR China; d Shanghai Tongji University affiliated Tongji Hospital, No. 389 Xinchun Road, Shanghai 200065, PR China; e Nanjing Brain Hospital, No. 264 Guangzhou Road, Nanjing, Jiangsu, 210029, PR China; f No. 4 Affiliated Hospital of Jiangsu University, No. 246 Nan Men Da Street, Zhenjiang, Jiangsu, 212001, PR China; g Second Affiliated Hospital of Zhejiang Chinese Medical University, No. 318 Chao Wang Road, Hangzhou, Zhejiang, 310005, PR China; h Tianjin Anding Hospital, No. 13 Liu Lin Road, Hexi District, Tianjin, 300222, PR China; i Shandong Mental Health Center, No. 49 East Wenhua Road, Jinan, Shandong, 250014, PR China; j No. 1 Hospital of Medical College of Xian Jiaotong University, No. 277 West Yan Ta Road, Xi'an, Shaanxi, 710061, PR China; k No. 1 Hospital of Zhengzhou University, No. 1 East Jianshe Road, Zhengzhou, Henan, 450052, PR China; l No. 1 Mental Health Center Affiliated Harbin Medical University, No. 23 You Zheng Jie, Nangang District, Harbin, Heilongjiang, PR China; m Mental Health Center of West China Hospital of Sichuan University, No. 28 Dian Xin Nan Jie, Wu Hou District, Chengdu, Sichuan 610041, PR China; n Beijing Anding Hospital, Capital Medical University, No. 5 An Kang Hutong Deshengmen wai, Xicheng District, Beijing, 100088, PR China; o Hebei Mental Health Center, No. 572 Dongfeng Road, Baoding, Hebei, 071000, PR China; p Shengjing Hospital of China Medical University, No. 36 Sanhao Street, Heping District Shenyang, Liaoning, 110817, PR China; q Shenzhen Kangning Hospital, No. 1080, Cui Zu Street, Luo Hu, Shenzhen, Guangdong, 518020, PR China; r No. 3 Affiliated Hospital of Sun Yat-sen University, No. 600 Tian He Road, Tian He District, Guangzhou, Guangdong, 510630, PR China; s No. 1 Hospital of Shanxi Medical University, No. 85 Jiefang South Road, Taiyuan, Shanxi, 030001, PR China; t Mental Hospital of Jiangxi Province, No. 43 Shangfang Road, Nanchang, Jiangxi, 330029, PR China; u The First Affiliated Hospital of Jinan University, No. 613 West Huangpu Avenue, Guangzhou, 510630, PR China; v Wuhan Mental Health Center, No. 70, You Yi Road, Wuhan, 430022, PR China; w No. 3 Hospital of Heilongjiang Province, No. 135 Jiao Tong Lu, Beian, Heilongjiang, PR China; x Jilin Brain Hospital, No. 98 Zhong Yang Xi Lu, Siping, Jilin, 136000, PR China; y The First Hospital of China Medical University, No. 155 Nanjing Bei Jie, He Ping District, Shenyang, Liaoning, 110001, PR China; z Dalian No. 7 People's Hospital & Dalian Mental Health Center, No. 179 Ling Shui Lu, Gan Jing Zi District, Dalian, Liaoning, PR China; aa The First Hospital of Hebei Medical University, No. 89 Donggang Road, Shijiazhuang, Hebei, 050031, PR China; ab Lanzhou University Second Hospital, Second Clinical Medical College of Lanzhou University, No. 82, Cui Ying Men, Lanzhou, Gansu, 730030, PR China; ac Psychiatric Hospital of Henan Province, No. 388 Jian She Zhong Lu, Xinxiang, Henan, PR China; ad The Fourth Military Medical University affiliated Xijing Hospital, No. 17, Changle West Road, Xi'an, Shaanxi, 710032, PR China; ae No. 4 People's Hospital of Liaocheng, No. 47 Hua Yuan Bei Road, Liaocheng, Shandong, 252000, PR China; af Guangzhou Brain Hospital/Guangzhou Psychiatric Hospital, No. 36 Ming Xin Lu, Fang Cun Da Dao, Li Wan District, Guangzhou, Guangdong, 510370, PR China; ag Clinical Trial Service Unit, Richard Doll Building, Old Road Campus, Roosevelt Drive, Oxford, OX3 7LF, UK; ah Virginia Commonwealth University, Department of Psychiatry, Virginia Institute for Psychiatric and Behavioral Genetics, Richmond, VA 23298-0126, USA

**Keywords:** Major depressive disorder, Age at onset, Symptom, Comorbidity

## Abstract

**Background:**

Individuals with early-onset depression may be a clinically distinct group with particular symptom patterns, illness course, comorbidity and family history. This question has not been previously investigated in a Han Chinese population.

**Methods:**

We examined the clinical features of 1970 Han Chinese women with DSM-IV major depressive disorder (MDD) between 30 and 60 years of age across China. Analysis of linear, logistic and multiple logistic regression models was used to determine the association between age at onset (AAO) with continuous, binary and discrete characteristic clinical features of MDD.

**Results:**

Earlier AAO was associated with more suicidal ideation and attempts and higher neuroticism, but fewer sleep, appetite and weight changes. Patients with an earlier AAO were more likely to suffer a chronic course (longer illness duration, more MDD episodes and longer index episode), increased rates of MDD in their parents and a lower likelihood of marriage. They tend to have higher comorbidity with anxiety disorders (general anxiety disorder, social phobia and agoraphobia) and dysthymia.

**Conclusions:**

Early AAO in MDD may be an index of a more severe, highly comorbid and familial disorder. Our findings indicate that the features of MDD in China are similar to those reported elsewhere in the world.

## 1 Introduction

The relationship between major depressive disorder (MDD) and variation in age at onset (AAO) has been frequently investigated both in community and treated samples studied in Europe and the US. Prior reports show that individuals with early-onset depression may be clinically distinct from late-onset depression with more symptoms (Gollan et al., 2005), more irritability and anxiety (Parker et al., 2003), higher rates of recurrence, longer episodes (Klein et al., 1999; Parker et al., 2003; Zisook et al., 2004) and greater familial loading (Kendler et al., 2005; Tozzi et al., 2008). However, studies have adopted different age-cut-offs to divide early- from late-onset forms (Corruble et al., 2008; Gallagher et al., 2009; Gollan et al., 2005; Parker et al., 2003) and few studies have investigated large samples (Corruble et al., 2008; Tozzi et al., 2008), perhaps explaining the discrepancies between studies (Brodaty et al., 2001; Klein et al., 1999; Zisook et al., 2004). Therefore large samples are required to clarify the relationship between AAO and indicators such as familial risk because of the relatively modest effect sizes so far identified (Kendler et al., 2005).

Comorbidity is common in MDD patients, with the commonest comorbid conditions being generalized anxiety disorder (GAD), panic disorder and dysthymia (Kessler et al., 2008). Although a number of studies have explored comorbidity of MDD (Angst, 1993; Merikangas et al., 1996; Pini et al., 1997; Tsuchiya et al., 2009), few have related the AAO of MDD to patterns of comorbidity (Alpert et al., 1999).

We examined the relationship between AAO and MDD in 1970 women with recurrent MDD recruited from hospitals across China. Given the expected relatively modest effects, we hypothesized that this large well-characterized sample might be sufficient to detect relationships between AAO and both the clinical features of MDD (such as lost of interest, melancholic features, suicidal ideation, illness course, comorbid psychiatric illness, etc.) and family history. Based on the literature and our previous work, we predicted that early- and late-onset MDD would be distinguishable both clinically and by familial loading. We predict that earlier AAO will generally reflect a greater severity of depressive illness as manifest throughout the life span.

## 2 Methods

### 2.1 Subjects

Data for the present work draw upon the ongoing China, Oxford and VCU Experimental Research on Genetic Epidemiology (CONVERGE) study of MDD. Analyses were based on a total of 1970 cases recruited from 53 provincial mental health centers and psychiatric departments of general medical hospitals in 41 cities in 19 provinces and four central cities: Beijng, Shanghai, Tianjin and Chongqing; 2597 controls were recruited from patients undergoing minor surgical procedures at general hospitals or from local community centers. All cases were female and had four Han Chinese grandparents. They were aged between 30 and 60, had suffered two or more episodes of MDD, with the first episode occurring between the ages of 14 and 50 and had not abused drugs or alcohol before their first episode of MDD. Cases were excluded if they had a pre-existing history of bipolar disorder, any type of psychosis or mental retardation.

All subjects were interviewed using a computerized assessment system, which lasted on average two hours for a case and one hour for a control. All interviewers were trained by the CONVERGE team for a minimum of one week in the use of the interview. The interview includes assessment of psychopathology, demographic and personal characteristics, and psychosocial functioning. Interviews were tape-recorded and a proportion of them were listened to by the trained editors who provided feedback on the quality of the interviews.

The study protocol was approved centrally by the Ethical Review Board of Oxford University and the ethics committee in participating hospitals in China.

### 2.2 Measures

The diagnoses of MDD, dysthymia, GAD and panic disorder were established with the Composite International Diagnostic Interview (CIDI, WHO lifetime version 2.1; Chinese version), which classifies diagnoses according to the Diagnostic and Statistical Manual of Mental Disorders (DSM-IV) criteria (American Psychiatric Association, 1987). Phobias, divided into five subtypes (animal, situational, social, blood-injury and agoraphobia) were diagnosed using an adaptation of DSM-III criteria (American Psychiatric Association, 1987) requiring one or more unreasonable fears and respondents' life being objectively affected by the symptoms. The section on the assessment of phobias was adopted from the interview used in the Virginia Adult Twin Study of Psychiatric and Substance Use Disorders (Kendler and Prescott, 2006). The history of lifetime MDD in the parents and siblings was assessed using the Family History Research Diagnostic criteria (Endicott et al., 1975). Neuroticism was measured with the 23-item Eysenck Personality Questionnaire (Eysenck and Eysenck, 1975). AAO of MDD was assessed retrospectively and defined as the age at which the first manifestation of MDD occurred, as reported by the participants. Symptoms are regarded as positive and were included in the analyses if they were reported to be present in the worst MDD episode. Anxiety was defined as the presence of unnecessary worries lasting either for one or six months'. Two types of positive family history were defined: (1) Type 1: one of the first degree family members (including parents and siblings) had a history of MDD. (2) Type 2: if one or both parents had a history of MDD.

Both the case and control interview were fully computerized into a bilingual system of Mandarin and English developed in house in Oxford, and called SysQ. Skip patterns were built into SysQ. Interviews were administered by trained interviewers and entered offline in real time onto SysQ, which was installed in the laptops. Once an interview was completed, a backup file containing all the previously entered interview data could be generated with database compatible format. The backup files together with an audio recording of the entire interview were uploaded to a designated server currently maintained in Beijing by a service provider. All the uploaded files in the Beijing server were then transferred to an Oxford server quarterly.

### 2.3 Statistical analysis

Statistical analyses were performed using the software package SPSS 17.0 (SPSS Inc., Chicago, IL). Descriptive statistics are presented as percentages for discrete variables and as means (and standard deviation) for continuous variables. Rather than using an age cut-off to distinguish early-late onset MDD, we used regression models to examine the association of AAO, treated as a continuous independent variable, with features of MDD. Linear and logistic regression models were used to determine the independent association of AAO with phenotypes. Coefficient values, odds ratios and 95% confidence intervals were used to quantify the strength of associations. The statistical significance for all tests was set at P < 0.05 as the analyses were exploratory in nature.

Symptoms reported during the most severe MDD episode were classified according to DSM-IV diagnostic “A criteria” such as lost of interest, weight loss/gain, insomnia/hypersomnia, etc. When testing the association between AAO and the number of MDD episodes, subjects who had “too many episodes to count” (n = 96) were excluded. Also when analyzing the longest episode in the patient's life so far, subjects who experienced episodes lasting longer than 480 weeks were excluded. When data were re-analyzed using the original data including these excluded subjects, the results of the analyses did not differ.

## 3 Results

The average age at interview was 45.1 (S.D. = 8.8) years (range 30–60). The mean age of MDD onset was 35.9(S.D. = 10.0) years (range 13–59). We analyzed the sample by logistic regression and estimated the effect of AAO by an odds ratio (OR). Table 1 shows the results for socio-demographic features and family history. Patients with an earlier AAO of MDD were likely to have received more education and be employed, but less likely to be married. We found a trend towards significance for family history (Type 1 in Table 1). We explored this further. We redefined family history as positive only if one or both parents were recorded as having an episode of MDD (Type 2 in Table 1). We found a significant inverse relationship (P = 0.002) with an OR of 0.98 per year of age.

Table 2 gives the association between AAO and the illness course and neuroticism of MDD patients. Earlier AAO was significantly associated with longer duration of MDD, more episodes, longer duration of the longest MDD episode and higher levels of the personality trait of neuroticism. No significant association was found between AAO and the number of symptoms experienced during the most severe MDD episode in patients' lifetime.

As the patients' current age could potentially influence duration of the illness and the number of episodes in lifetime, we analyzed the association of AAO with current age, illness course and episode number respectively. The results showed that patients with an earlier AAO were younger. However, age was not a confounding factor in the association of AAO with duration and number of episodes; the magnitude of the association did not change substantially when age was added as a covariate.

Table 3 summarizes the association between AAO, individual symptoms during the worst MDD episode and lifetime risk for other co-morbid psychiatric disorders. Earlier AAO was positively associated with suicidal ideation or attempt while negatively associated with changes in sleep or appetite/weight. Subjects with an earlier AAO for MDD were at significantly higher risk for GAD, agoraphobia, social phobia and dysthymia.

## 4 Discussion

Our analyses uncovered five major differences between subjects with an early versus later onset of MDD. We review each of these results in turn, putting them in the context of the relevant prior literature. First, patients with earlier AAO are more likely to be burdened with a severe and chronic mood disorder. This is consistent with the Western literature in which an earlier AAO has a more malignant course, characterized by more MDD episodes and a longer index MDD episode (Birmaher et al., 1996; Glied and Pine, 2002; Gollan et al., 2005; Klein et al., 1999; Lewinsohn et al., 1994; Parker et al., 2003; Zisook et al., 2004, 2007). Our results are in agreement that MDD of early onset, at least in Chinese women, is more severe than MDD of later onset.

Second, we find that cases with an earlier AAO of MDD have a relatively distinct clinical picture, with reduced levels of more biological symptoms. In part, the symptoms we identify may reflect greater severity (increased rates of suicide ideation and attempts) but they may also indicate the existence of a characteristic symptom profile. In this regard there is less agreement among the extant literature, which reports that earlier AAO of MDD both is (Zisook et al., 2004, 2007), and is not (Brodaty et al., 2001; Klein et al., 1999), associated with greater symptom severity. Parker and colleagues argue that depressive symptoms in early AAO patients do not mark them out (Parker et al., 2003), but that there may be a distinct temperamental dimension. As noted below, this may reflect increased rates of neuroticism, which we detected in our sample.

Third, earlier AAO for MDD is associated with greater rates of co-morbid anxiety disorders and higher rates of neuroticism. Reports in the literature have shown that earlier AAO of MDD does confer greater risk of psychiatric co-morbid illness, including GAD, panic disorder, phobia, dysthymia (Alpert et al., 1999; Gabilondo et al., 2010; Parker et al., 2003) and higher scores of neuroticism (Tozzi et al., 2008). Early onset subjects may be distinguished by a greater irritability, reflecting increased emotional reactivity, or neuroticism (Parker et al., 2003). This would be consistent with the increased rates of neuroticism we observed. It should be noted that the absolute value of neuroticism scores is lower in our sample, compared to those reported in Western studies (Tozzi et al., 2008), but that the relative difference, between patients and controls is comparable.

Fourth, we found that patients with an earlier AAO of MDD were more likely to be single. Given the higher rates of psychiatric comorbidity and chronicity of MDD, it might be surmised that earlier AAO gives rise to more impaired social and occupational function, which in turn is associated with lower educational attainment and likelihood of marriage. Results from one US study support such a view. Analysis of 4041 participants enrolled in a study of MDD treatment (Zisook et al., 2004, 2007) found earlier AAO to be associated with significant functional impairment, worse illness (more severe depressive episodes and greater suicidal ideation), greater psychiatric comorbidity and a higher likelihood to be “never married”. However, this cannot fully explain findings in our cohort because our patients with an earlier AAO of MDD were more likely to have received more education and to be employed. To help understand this inconsistency we should take account of socio-cultural differences between China and the West. While most of our patients grew up during the 1970s, and have less education, patients with an earlier AAO of MDD in our sample tend to be younger. They were therefore born later, and grew up during the 1980s when China's reforms had been implemented, and higher education more available.

Fifth, our findings add to the literature about the disputed relationship between AAO and family history (Lyons et al., 1998; Sullivan et al., 2000; Tozzi et al., 2008; Weissman et al., 1986). We found a trend towards significance for family history (Type 1 in Table 1), but this only became significant when we defined family history as MDD in one or both parents (Type 2 in Table 1). We suspect that information about the history of MDD in parents is more reliable than that in siblings because all parents of our patients would have completed their age at risk for MDD but this would not be the case for all siblings. Consequently including information about siblings will tend to reduce power because it increases the amount of false negatives. Our finding suggests that the effect, if present, may be relatively small, a view that accords with results in the Western literature. Notably, while the first 1500 MDD patients from a large cohort failed to find earlier AAO associated with family history (Zisook et al., 2004), analysis of the next 2500 MDD patients did find a relationship (Zisook et al., 2007). Furthermore, a well powered study of 13,864 twins concluded that AAO does have a modest negative relationship to the risk of illness in relatives and this relationship is not linear: it is undetectable in those with an onset age of 35 years or more (Kendler et al., 2005). We need a more fine-grained analysis in a larger sample to determine the relationship between family history and AAO.

Several limitations of this study should be considered. First, this is a cross sectional study: data were collected retrospectively and recall bias will have affected results. Second, all of the patients were from in-patient hospital samples so our findings are not representative of community samples (which are likely to include less severely ill patients). Third, all of the patients were female, so applicability to male patients is unknown. Other limitations include not interviewing directly other family members and not assessing the inter-rater reliability of the interview.

In summary, Chinese women with an earlier AAO of MDD have some distinct symptom patterns, more chronic course, higher psychiatry comorbidity rates and higher family history of MDD. Earlier AAO may indicate a more malignant, chronic condition with higher genetic loading that should be recognized by clinicians in their daily practice. Our findings parallel those reported in the West and add to the literature indicating that the features of MDD in China are similar to those reported elsewhere in the world.

## Role of funding source

Funding for this study was provided by the Wellcome Trust; the Wellcome Trust had no further role in study design; in the collection, analysis and interpretation of data; in the writing of the report; and in the decision to submit the paper for publication.

## Conflict of interest

All authors declare they have no conflicts of interest including any financial, personal or other relationships with other people or organizations within three years of beginning the work submitted that could inappropriately influence, or be perceived to influence, their work.

## Figures and Tables

**Table 1:** Association of MD age at onset of MDD with socio-demographic variables and family history.

Variable	Number	Number (%)	OR (95% CI)	P*-value*
Marital status	1919			
Married		1606 (83.7%)		
Separated		38 (2.0%)	0.98 (0.95–1.01)	0.244
Divorced		164 (8.5%)	0.98 (0.96–0.99)	0.008
Widowed		64 (3.3%)	1.04 (1.01–1.07)	0.004
Never married		47 (2.5%)	0.91 (0.88–0.94)	< 0.0001
Education	1915			
Primary school		391 (20.4%)		
Middle school		865 (45.2%)	1.00 (0.99–1.01)	0.827
Technical school		206 (10.8%)	0.99 (0.97–1.01)	0.254
College/graduate		453 (23.6%)	0.94 (0.93–0.96)	< 0.0001
Occupation	1915			
Unemployed		1171 (61.1%)		
Employed		601 (31.4%)	0.94 (0.93–0.95)	< 0.0001
Other		143 (7.5%)	0.94 (0.93–0.96)	< 0.0001
Family history Type 1	1881			
No		1265 (67.3%)		
Yes		616 (32.7%)	0.99 (0.98–1.00)	0.104
Family history Type 2	1555			
No		1173 (75.4%)		
Yes		382 (24.6%)	0.98 (0.97–0.99)	0.002

OR odds ratio; CI confidence interval; MDD major depressive disorder.

**Table 2:** Association of MDD age at onset with clinical features of MDD and neuroticism.

Characteristic	N	Mean (S.D.)	Coefficient (95% CI)	P-value
Age	1912	45.06 (8.83)	0.57 (0.54–0.60)	< 0.0001
Length of illness course	1854	9.46 (7.93)	− 0.42 (− 0.46–0.40)	< 0.0001
Number of episodes	1892	4.20 (5.03)	− 0.06 (− 0.08–0.04)	< 0.0001
Length of longest episode	1894	38.03 (52.50)	− 0.41 (− 0.65–0.17)	0.001
Number of symptoms in the most severe episode	1921	8.30 (1.01)	0.002 (− 0.002–0.007)	0.332
Neuroticism score	1878	12.72 (5.83)	− 0.11 (− 0.13–0.08)	< 0.0001

Results given are from linear regression. Neuroticism scores are standardized. N: number; SD standard deviation; CI confidence interval; MDD major depressive disorder.

**Table 3:** Association of MD age at onset of MDD with MDD symptoms and co-morbid diseases.

Symptoms and axis I illness	Number (%)	OR (95% CI)	P-value
Loss of interest	1886 (98.2)	1.01 (0.97–1.04)	0.68
Weight loss/gain, appetite changes	1750 (90.9)	1.03 (1.02–1.05)	< 0.0001
Insomnia/hypersomnia	1838 (95.5)	1.02 (1.00–1.05)	0.037
Psychomotor agitation/retardation	1725 (89.6)	1.01 (1.00–1.03)	0.095
Fatigue/loss of energy	1792 (93.1)	1.02 (1.00–1.03)	0.10
Feelings of worthlessness/guilt	1723 (89.5)	0.99 (0.97–1.00)	0.11
Concentrate/indecisiveness	1876 (97.5)	0.99 (0.96–1.01)	0.32
Suicidal ideation/attempt	1463 (76.0)	0.99 (0.98–0.99)	0.02
GAD	569 (29.9)	0.98 (0.97–0.99)	< 0.0001
Panic	192 (10.1)	1.00 (0.99–1.02)	0.90
Dysthymia	344 (17.9)	0.96 (0.95–0.97)	< 0.0001
Agoraphobia	500 (26.6)	0.99 (0.98–0.99)	0.031
Social phobia	598 (31.9)	0.99 (0.98–0.99)	0.022
Blood phobia	747 (39.8)	0.99 (0.99–1.02)	0.078
Animal phobia	1043 (55.6)	0.99 (0.99–1.00)	0.26
Situational phobia	736 (39.2)	0.99 (0.99–1.00)	0.30

OR odds ratio; CI confidence interval; MDD major depressive disorder; GAD generalized anxiety disorder.
